# Editorial: Case reports in respiratory pharmacology 2022

**DOI:** 10.3389/fphar.2023.1242273

**Published:** 2023-08-15

**Authors:** Przemyslaw Zdziarski, Luisa Ricciardi, Roberto Paganelli

**Affiliations:** ^1^ Department of Clinical Immunology and Pulmonary Disease, Lower Silesian Oncology, Pulmonology and Hematology Centre, Wroclaw, Poland; ^2^ Department of Clinical and Experimental Medicine, University of Messina, Messina, Italy; ^3^ Internal Medicine, Saint Camillus International University of Medical and Health Sciences, Rome, Italy

**Keywords:** respiratory pharmacology, case study, patient-centered care, SARS-CoV-2, corticosteroids, pharmacovigilance, eosinophilic granulomatosis with polyangiitis (EGPA), off-label

Despite the separation of pulmonology from other disciplines, the diseases of the respiratory system may present systemic symptoms and their treatment cause systemic effects. Tuberculosis and SARS, atopic asthma, lymphoid interstitial pneumonia, Eosinophilic Granulomatosis with Polyangiitis (EGPA), and immunoendocrinopathy, described in our Research Topic, are good examples. On the other hand, this makes construction of cohort studies more difficult. Lessons from individual clinical cases with a comprehensive, holistic approach can be all the more instructive, especially in rare diseases or long-term observation (Table 1).

**TABLE 1:** Complementarity of cohort and case studies.

Studies	Cohort	Case reports	Example (from the Research Topic)
Area			
Study of rare diseases or rare constellations	impractical	useful	Wang et al.
			Ning et al.
Application in the clinic and treatment strategy	indirect	direct	Ogletree et al.
			Ricciardi et al.
Nature of the study	prospective	retrospective	all listed
Comprehensive description of natural history of disease	No	Yes	Wang et al. *(myxedema)*
			Ricciardi et al. (EGPA)
Time-line of therapeutic regimens with many drugs	No	Yes	Wang et al.
			Ricciardi et al.
Risk factors analyzed	limited	multiple	Ricciardi et al. *(population at risk)*
Cause-effect relationship (association between exposure and an event)	Statistical correlation	Temporal, pathogenetic links	Ogletree et al.
			Ning et al.
			Ricciardi et al. *(circumstantial)*
Adverse drug reaction and causality assessment^a^	Must be planned (usually predictable ARDs)	Simple (e.g., based on drug withdrawal)	Ning et al.

The table shows the areas of knowledge reserved for cohort or case studies, indicating strengths/weaknesses as well as examples within the article collection (Frontiers in Pharmacology Research Topic). Case studies give the possibility of collecting all data (for patient-centered care), instead of those used for planning prospective studies.

^a^ Unpredictable side effects (also beneficial) are the issue that cannot be directly planned in cohort studies.

This Research Topic included four high-quality case reports published last year in Frontiers in Pharmacology, highlighting aspects of clinical pharmacology of drugs used for the treatment of respiratory diseases. The description of the diagnostic work-up, clinical assessment, therapeutic measures and monitoring of outcomes of single cases or small series of patients is a very useful adjunct to large clinical trials, particularly when evaluating uncommon conditions, such as those presented here: muscle paralysis, myxedema, or the non-standard (off-label) use of approved drugs (e.g., ramatroban and benralizumab in our series, see below). These descriptions represent examples of personalized medicine, since standard guidelines are missing or not applicable (Table 1).

Wang et al. described how overlooking untreated hypothyroidism on hospital admission may lead to severe respiratory distress. The role of hypothyroidism in cardiology is well known (Kagansky et al., 2023). Although cardiomegaly (primarily due to pericardial effusion) is a typical sign of myxedema, it develops slowly, rarely causing hemodynamic distress (Glenn and Braunstein, 2022); therefore, it may go unnoticed for a long time. Currently, when subclinical hypothyroidism is diagnosed, its late-stage forms, such as myxedema, are often underestimated, because dyspnea suggests cardiopulmonary disorders. In the study, a delayed and inadequate initial treatment with low T4 dose was adopted, thus prolonging edema and causing multi-organ failure. This report also advocates the use of glucocorticoid treatment in addition to adequate dose of i. v. levothyroxine (Glenn and Braunstein, 2022), since T4 increases glucocorticoid receptors expression, facilitating steroid anti-edematous effects through an increased diuretic action (Liu et al., 2006). The inclusion of steroids in the treatment may be beneficial by interacting with the hypothalamus-pituitary- adrenal axis (decrease of ACTH and corticotropin-releasing hormone - direct inhibitor of TSH secretion), as well as by inhibiting pathogenic IgG autoantibodies synthesis (Zdziarski et al., 2022).

Pulmonary infections, especially with viral pathogens, are considered among the leading causes of mortality, fast pandemic spread and high economic costs. The efficacy of Ramatroban, a dual Thromboxane A2 and Prostaglandin D2 receptor antagonist, in COVID-19 pneumonia was described by Ogletree et al. in four cases whose improvement avoided hospitalization despite initial respiratory distress. The possible mechanisms ranged from improved ventilation-reperfusion matching, to restored type 1 Interferon production at epithelial surfaces. Moreover, no long-term sequelae of COVID-19, such as lung fibrosis (Kimura et al., 2023), were observed.

The treatment of bacterial respiratory infections in the era of antibiotic resistance is complicated also by adverse drug reactions (ADRs). An estimated 5%–25% of hospital admissions are due to ADRs, and 6%–15% of hospitalized patients experience serious ADRs (source VigiAccess–Adverse Drug Reaction (ADR) Database—World Health Organization’s free database), causing significant prolongation of hospital stay (Ramirez et al., 2022). Ning et al. reported respiratory muscle paralysis in a transplant patient treated with Polymixin B, a rare but possibly fatal complication of this drug, known for its potential nephrotoxicity (Sorli et al., 2013) and risk of anaphylaxis (Zhan et al., 2019). This life threatening ADR should alert doctors treating patients with renal dysfunctions.

A fourth case report dealt with the treatment of a patient with EGPA experiencing severe asthma Ricciardi et al. The patient was a difficult case, diagnosed only after several episodes of pericarditis, with anti-neutrophil cytoplasmic antibodies (ANCA) resulting negative, and also considering her young age (22 years). Since oral corticosteroids had minimal effects, she was treated with an anti-IL-5R, benralizumab, not approved for EGPA (off-label), at variance with the anti-IL5 monoclonal mepolizumab (Koike et al., 2023). A rapid benefit was observed, but the interest of this case lies in the recognition of the pitfalls when diagnosing unusual presentations: also ANCA-negative patients with early-onset asthma should be investigated for EGPA in future studies.

These four reports illustrate some important points in the field, such as the use of corticosteroids in severe hypothyroidism, the possible use of benralizumab in systemic vasculitis, and of ramatroban in COVID-19 (Kupczyk and Kuna, 2017; Al-Kuraishy et al., 2023). They also show persisting shadows in the pharmacovigilance of colistin, and pitfalls in diagnosing emergency admissions, such as overlooking untreated hypothyroidism.

Unusual clinical aspects and responses to treatment in this series may help personalized and more rational pharmacological therapy of respiratory diseases, with the identification of pertinent outcomes. The case studied may help clinicians choose therapeutic options in other rare conditions.

## Abbreviations

ADRs, Adverse drug reactions; ARD, Acute Respiratory Distress; ACTH or corticotropin, Adrenocorticotropic hormone; EGPA, Eosinophilic Granulomatosis with Polyangiitis; SARS-CoV-2, Severe Acute Respiratory Syndrome Coronavirus-2; TV, Tidal volume.
